# Real-world incidence of cancer therapy–related cardiac dysfunction in a large, diverse, and contemporary cohort

**DOI:** 10.1093/eschf/xvag113

**Published:** 2026-04-30

**Authors:** Samir R Thadani, Alan S Go, Jane Y Liu, Rishi V Parikh, Elisha A Garcia, Elizabeth M Cespedes Feliciano, Ankeet S Bhatt, Sirtaz Adatya, Marilyn L Kwan, Amy Y Lin, Raymond Liu, Alfredo Lopez, Joshua R Nugent, David Ouyang, Alberta H Yen, Jonathan G Zaroff, Andrew P Ambrosy

**Affiliations:** Department of Cardiology, Kaiser Permanente South SanFrancisco Medical Center, 1200 El Camino Real, South San Francisco, CA 94080, USA; Division of Research, Kaiser Permanente Northern California, Pleasanton, CA, USA; Department of Health Systems Science, Kaiser Permanente Bernard J. Tyson School of Medicine, Pasadena, CA, USA; Departments of Epidemiology, Biostatistics and Medicine, University of California, SanFrancisco, San Francisco, CA USA; Division of Research, Kaiser Permanente Northern California, Pleasanton, CA, USA; Division of Research, Kaiser Permanente Northern California, Pleasanton, CA, USA; Division of Research, Kaiser Permanente Northern California, Pleasanton, CA, USA; Division of Research, Kaiser Permanente Northern California, Pleasanton, CA, USA; Division of Research, Kaiser Permanente Northern California, Pleasanton, CA, USA; Department of Cardiology, Kaiser Permanente SanFrancisco Medical Center, San Francisco, CA, USA; Department of Cardiology, Kaiser Permanente Santa Clara, Santa Clara, CA, USA; Division of Research, Kaiser Permanente Northern California, Pleasanton, CA, USA; Department of Cardiology, Kaiser Permanente SanFrancisco Medical Center, San Francisco, CA, USA; Department of Cardiology, Kaiser Permanente SanFrancisco Medical Center, San Francisco, CA, USA; Department of Cardiology, Kaiser Permanente SanFrancisco Medical Center, San Francisco, CA, USA; Division of Research, Kaiser Permanente Northern California, Pleasanton, CA, USA; Division of Research, Kaiser Permanente Northern California, Pleasanton, CA, USA; Department of Cardiology, Kaiser Permanente Santa Clara, Santa Clara, CA, USA; Department of Cardiology, Kaiser Permanente Santa Clara, Santa Clara, CA, USA; Department of Cardiology, Kaiser Permanente SanFrancisco Medical Center, San Francisco, CA, USA; Division of Research, Kaiser Permanente Northern California, Pleasanton, CA, USA; Department of Cardiology, Kaiser Permanente SanFrancisco Medical Center, San Francisco, CA, USA

**Keywords:** Chemotherapy, Cardio-oncology, Cancer therapy–related cardiac dysfunction, Heart failure

## Abstract

**Background and Aims:**

Cancer therapy–related cardiac dysfunction (CTRCD) is a complication of contemporary oncologic treatment and a contributor to incident heart failure (HF) in cancer survivors. Although certain potentially cardiotoxic cancer therapies are known to increase risk, population-based estimates in large, diverse, and contemporary cohorts remain limited. The aim of the Kaiser Permanente Cardiovascular Health Enhancement and Monitoring for Oncology (KP CHEMO) study was to determine the incidence, timing, and treatment-specific variation in CTRCD within an integrated US health system.

**Methods and Results:**

We conducted a retrospective cohort study of adult Kaiser Permanente Northern California (KPNC) members diagnosed with malignant tumours between 2012 and 2022 who received anthracyclines, human epidermal growth factor receptor (HER2) inhibitors, immune checkpoint inhibitors (ICIs), or tyrosine kinase inhibitors. CTRCD was defined as a >10% decline in left ventricular ejection fraction to <53% or incident HF identified by natural language processing. Cumulative incidence rates were calculated overall and by drug class. Early CTRCD was ≤12 months and late was >12 months. Among 26 646 patients (mean age 62 ± 14 years; 64% women; 57% non-Hispanic White), the cumulative incidence of CTRCD was 8.4% (95% confidence interval 7.7–9.1). Incidence was highest with HER2 inhibitors (10.7%) and lowest with ICIs (5.2%) (*P* < .001). Nearly half of all events occurred within the first year.

**Conclusions:**

CTRCD was common and occurred predominantly within the first year after therapy initiation, potentially reflecting both early susceptibility and more intensive early surveillance. Variation across drug classes highlights differing cardiotoxic risk profiles. These findings support risk prediction models and targeted surveillance strategies to reduce downstream HF risk.

## Background

Approximately 19 million cancer survivors currently live in the USA.^1^ With longer lifespans from expanding cancer therapies, the burden of cardiovascular complications—including cancer therapy–related cardiac dysfunction (CTRCD)—is increasing. Observational studies suggest up to 10% of patients treated with certain chemotherapeutic agents develop CTRCD, which historically was associated with adverse outcomes, although more recent data suggest a heterogeneous clinical course with potential for recovery in some patients.^2–5^ While specific agents such as anthracyclines and human epidermal growth factor receptor (HER2) inhibitors have well-known cardiotoxic potential, contemporary real-world estimates of CTRCD in diverse populations are limited. Most available data originate from clinical trials or single-centre cohorts often focused on anthracyclines and HER2 inhibitors and may not reflect the broader patient population. Updated epidemiologic data are needed to inform surveillance strategies and guide risk stratification and intervention.

## Aims

The aim of the Kaiser Permanente Cardiovascular Health Enhancement and Monitoring for Oncology (KP CHEMO) study was to characterize the incidence, timing, and treatment-specific variation of CTRCD among adults receiving potentially cardiotoxic cancer therapies within a large, integrated US healthcare system, capturing multiple contemporary therapy classes in routine clinical practice. We sought to provide updated, real-world estimates to help inform surveillance strategies and guide future risk prediction efforts.

## Methods

We performed a retrospective cohort study of adult members of Kaiser Permanente Northern California (KPNC), a large integrated healthcare system serving over 4.5 million individuals. Eligible participants included those diagnosed with malignant solid or haematologic tumours (excluding non-melanoma skin cancer) between 1 January 2012 and 31 December 2022 who received at least one potentially cardiotoxic cancer therapy: anthracyclines, HER2 inhibitors, immune checkpoint inhibitors (ICIs), or tyrosine kinase inhibitors (TKIs). Continuous health plan enrolment of ≥6 months prior to cancer diagnosis was required. Patients with asymptomatic left ventricular (LV) dysfunction (LV ejection fraction [LVEF] <53%) or a heart failure (HF) diagnosis code prior to index date were excluded.

Data were obtained from the KPNC Cancer Registry, Virtual Data Warehouse, and electronic health record. The primary outcome was CTRCD, defined as either (i) LV dysfunction (>10% absolute decline from normal to LVEF <53%)^6^ or (ii) incident clinical HF identified using a validated natural language processing algorithm.^7,8^ This rule-based algorithm integrates structured data (e.g. diagnostic codes, laboratory values, medication use) with unstructured clinical text (e.g. physician notes, imaging reports) to identify clinical HF. LVEF measurements were extracted from transthoracic echocardiograms or multiple-gated acquisition scans. Baseline LVEF was defined as the closest value prior to or shortly following therapy initiation. Early events were defined as occurring ≤12 months after treatment initiation; late events occurred thereafter.

Patients were followed from therapy initiation until death, health plan disenrollment, or 31 December 2023. We calculated Aalen–Johansen cumulative incidence estimates for all CTRCD, HF events, and LV dysfunction with death as a competing risk, overall and stratified by drug class and cancer type. Differences between cumulative incidence curves were assessed using Gray’s test.

## Results

A total of 26 646 patients met inclusion criteria, with a mean age of 62 ± 14 years; 64% were women, and 57% identified as non-Hispanic White. The most common cancer types were breast (32%), lung (17%), and haematologic malignancies (15%) (*Table 1*). Exposure to potentially cardiotoxic cancer therapies was as follows in the cohort: 38% received anthracyclines, 15% HER2-targeted agents, 26% ICIs, and 22% TKIs. Prior medical history included 49% with dyslipidaemia, 46% with hypertension, and 26% with chronic lung disease or obstructive sleep apnoea.

**Table 1:** Baseline characteristics

Characteristic	Overall	Anthracyclines	HER2 inhibitors	ICIs	TKIs
	*N* = 26 646	*N* = 10 058	*N* = 3926	*N* = 6847	*N* = 5815
Age, years, mean (SD)	61.7 (14.0)	56.8 (14.3)	58.2 (12.6)	67.7 (11.9)	65.6 (12.9)
Female, *n* (%)	17 158 (64.4)	7699 (76.5)	3716 (94.7)	2968 (43.3)	2775 (47.7)
Race/ethnicity, *n* (%)					
American Indian or Alaska Native	73 (0.3)	33 (0.3)	5 (0.1)	22 (0.3)	13 (0.2)
Asian	5099 (19.1)	1818 (18.1)	872 (22.2)	982 (14.3)	1427 (24.5)
Hispanic	3230 (12.1)	1474 (14.7)	484 (12.3)	638 (9.3)	634 (10.9)
Native Hawaiian/Pacific Islander	178 (0.7)	83 (0.8)	24 (0.6)	38 (0.6)	33 (0.6)
Non-Hispanic White	15 209 (57.1)	5499 (54.7)	2090 (53.2)	4533 (66.2)	3087 (53.1)
Non-Hispanic Black	1829 (6.9)	744 (7.4)	279 (7.1)	417 (6.1)	389 (6.7)
Multi-racial	1009 (3.8)	400 (4.0)	165 (4.2)	217 (3.2)	227 (3.9)
Unknown	19 (0.1)	7 (0.1)	7 (0.2)	0 (0.0)	5 (0.1)
Current smoker, *n* (%)	1506 (5.7)	459 (4.6)	170 (4.3)	613 (9.0)	264 (4.5)
SEER stage, *n* (%)					
Localized	6614 (24.8)	2280 (22.7)	2059 (52.4)	1295 (18.9)	980 (16.9)
Regional by direct extension only	1935 (7.3)	855 (8.5)	45 (1.1)	552 (8.1)	483 (8.3)
Regional lymph nodes only	5235 (19.6)	2798 (27.8)	1190 (30.3)	984 (14.4)	263 (4.5)
Regional by direct extension and lymph nodes	1576 (5.9)	484 (4.8)	143 (3.6)	682 (10.0)	267 (4.6)
Regional, not specified	288 (1.1)	241 (2.4)	0 (0.0)	20 (0.3)	27 (0.5)
Distant site(s)/node(s)	10 608 (39.8)	3309 (32.9)	472 (12.0)	3132 (45.7)	3695 (63.5)
Unknown	390 (1.5)	91 (0.9)	17 (0.4)	182 (2.7)	100 (1.7)
Cancer type, *n* (%)					
Breast	8517 (32.0)	4621 (45.9)	3607 (91.9)	224 (3.3)	65 (1.1)
Lung	4595 (17.2)	59 (0.6)	19 (0.5)	2696 (39.4)	1821 (31.3)
Haematological malignancies	3937 (14.8)	2613 (26.0)	5 (0.1)	37 (0.5)	1282 (22.0)
Renal/urinary	1782 (6.7)	303 (3.0)	5 (0.1)	783 (11.4)	691 (11.9)
Gynaecological	1616 (6.1)	1174 (11.7)	26 (0.7)	266 (3.9)	150 (2.6)
Upper gastrointestinal	1411 (5.3)	416 (4.1)	212 (5.4)	519 (7.6)	264 (4.5)
Melanoma	1291 (4.8)	51 (0.5)	3 (0.1)	1104 (16.1)	133 (2.3)
Hepatobiliary	815 (3.1)	24 (0.2)	16 (0.4)	254 (3.7)	521 (9.0)
Lower gastrointestinal	695 (2.6)	25 (0.2)	18 (0.5)	214 (3.1)	438 (7.5)
Head and neck	517 (1.9)	104 (1.0)	3 (0.1)	377 (5.5)	33 (0.6)
Musculoskeletal	422 (1.6)	307 (3.1)	0 (0.0)	13 (0.2)	102 (1.8)
Other	1048 (3.9)	361 (3.6)	12 (0.3)	360 (5.3)	315 (5.4)
Liquid tumour, *n* (%)	3937 (14.8)	2613 (26.0)	5 (0.1)	37 (0.5)	1282 (22.0)
Prior medical history, *n* (%)					
Atrial fibrillation or flutter	1206 (4.5)	257 (2.6)	79 (2.0)	547 (8.0)	323 (5.6)
Ischaemic stroke or transient ischaemic attack	283 (1.1)	68 (0.7)	19 (0.5)	122 (1.8)	74 (1.3)
Myocardial infarction	76 (0.3)	16 (0.2)	4 (0.1)	33 (0.5)	23 (0.4)
Coronary artery disease	23 (0.1)	4 (0.0)	0 (0.0)	12 (0.2)	7 (0.1)
Peripheral artery disease	455 (1.7)	88 (0.9)	22 (0.6)	255 (3.7)	90 (1.5)
Valvular disease	466 (1.7)	121 (1.2)	54 (1.4)	173 (2.5)	118 (2.0)
Venous thromboembolism	1573 (5.9)	489 (4.9)	58 (1.5)	555 (8.1)	471 (8.1)
Diabetes mellitus	1128 (4.2)	396 (3.9)	153 (3.9)	257 (3.8)	322 (5.5)
Hypertension	12 342 (46.3)	3805 (37.8)	1519 (38.7)	3859 (56.4)	3159 (54.3)
Dyslipidaemia	12 936 (48.5)	4091 (40.7)	1587 (40.4)	4084 (59.6)	3174 (54.6)
Chronic liver disease	1894 (7.1)	503 (5.0)	169 (4.3)	531 (7.8)	691 (11.9)
Chronic lung disease or obstructive sleep apnoea	6833 (25.6)	2033 (20.2)	793 (20.2)	2505 (36.6)	1502 (25.8)

HER2, human epidermal growth factor receptor; ICI, immune checkpoint inhibitor; TKI, tyrosine kinase inhibitor; SD, standard deviation; SEER, surveillance, epidemiology, and end results.

**Figure 1:**
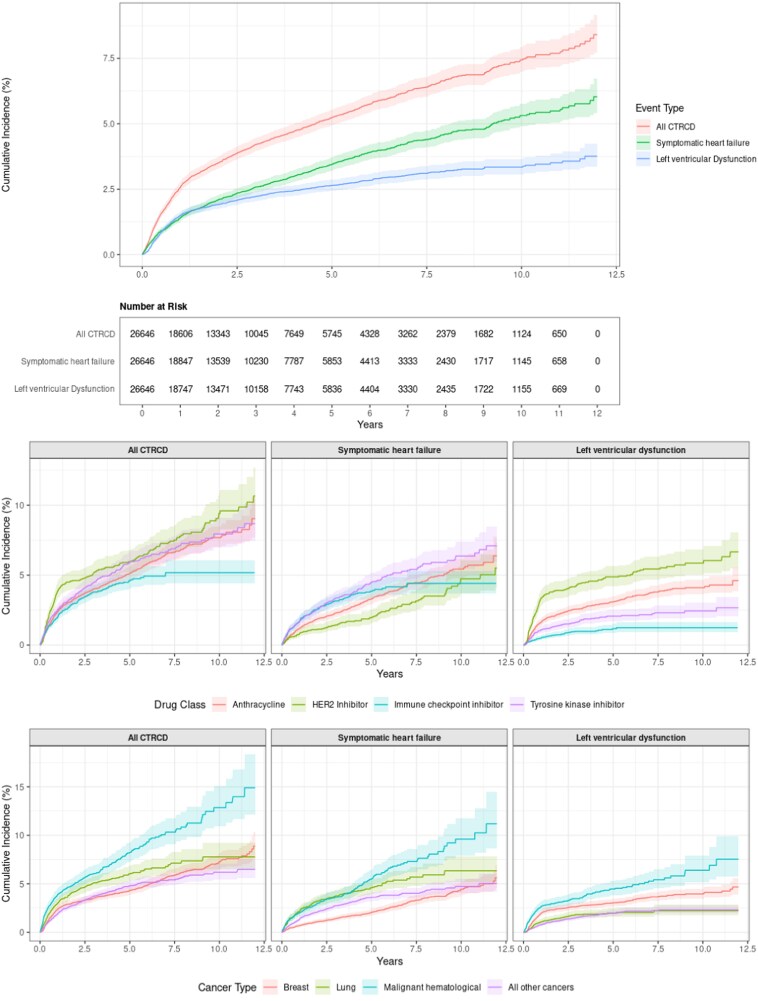
Overall incidence of cancer therapy–related cardiac dysfunction segmented by type of event as well as incidence of cancer therapy–related cardiac dysfunction by cancer drug class and cancer type in 26 646 adult patients receiving potentially cardiotoxic cancer therapies in Kaiser Permanente Northern California, 2012–22. Cancer therapy–related cardiac dysfunction is defined as asymptomatic left ventricular dysfunction (>10% absolute decline from normal to left ventricular ejection fraction <53%) or incident heart failure. HER2, human epidermal growth factor receptor

Over a maximum follow-up period of 12 years [median (Q1, Q3) follow-up 2.0 (0.7, 4.5) years], we observed 1342 CTRCD events corresponding to an overall 12-year cumulative incidence of CTRCD of 8.4% (95% confidence interval: 7.7, 9.2) (*Figure 1*). Among those who experienced CTRCD, 459 (34%) experienced asymptomatic LV dysfunction alone, 671 (50%) experienced symptomatic HF alone, and 212 (16%) experienced both HF and LV dysfunction. Incidence varied significantly by therapeutic class and cancer type (*P* < .001 for all) (*Figure 1*). Patients treated with HER2 inhibitors experienced the highest CTRCD incidence (10.7%). In contrast, patients receiving ICIs had the lowest incidence (5.2%) (*Figure 1*). When segmented by cancer type, those with haematological malignancies had the highest CTRCD cumulative incidence (14.9%).

Nearly half (49.9%) of all CTRCD events occurred within the first year following therapy initiation.

## Conclusions

In this large, diverse, and contemporary US cohort of patients with cancer, CTRCD was common, affecting nearly 1 in 10 individuals exposed to potentially cardiotoxic cancer therapies. The predominance of early events suggests that cardiotoxicity may manifest soon after therapy initiation, although this pattern may also reflect more intensive early surveillance and differential detection over time. Variation in incidence across drug classes aligns with known cardiotoxicity profiles and reinforces the importance of individualized surveillance based on therapeutic exposure.^9^

Limitations include inability to precisely attribute risk to specific cancers given overlapping treatments, as well as potential confounding from differing baseline cardiovascular risk profiles and surveillance intensity, as we did not perform stratified analyses across baseline cardiovascular risk factors. Accordingly, comparisons across treatment groups should be interpreted cautiously, as observed differences may reflect underlying patient characteristics and treatment selection rather than causal effects of specific therapies. Further stratified analyses across demographic and baseline cardiovascular risk factors may provide additional insights into heterogeneity of CTRCD risk. We also did not account for baseline or time-varying use of cardioprotective medications, which may influence CTRCD risk. Additionally, data on radiotherapy exposure were not available, and not all potentially cardiotoxic cancer therapies were captured (e.g. proteasome inhibitors used in haematologic malignancies such as multiple myeloma). For patients receiving multiple agents, toxicity was attributed to the first drug, which may oversimplify time-dependent or cumulative effects across sequential therapies; although time-varying exposure models could better account for treatment sequencing, they would introduce additional complexity and may limit interpretability in the context of overlapping therapies. Echocardiograms were obtained at provider discretion rather than at pre-specified intervals, which may introduce detection bias, including greater identification of events early after therapy initiation when surveillance is more frequent. Finally, our CTRCD definition, based on LVEF decline and/or incident HF, does not incorporate more sensitive markers such as global longitudinal strain or cardiac biomarkers and likely underestimates the true burden of CTRCD, primarily reflecting moderate-to-severe disease according to contemporary ESC cardio-oncology definitions.^5^

These findings provide real-world context for the burden and timing of CTRCD across multiple modern cancer therapies and highlight a critical need for validated short-term risk prediction models to identify patients at greatest risk for early cardiotoxicity. Integrating such tools into electronic health records could enable more efficient triage into risk-based surveillance pathways, while health system–level cardio-oncology programmes may mitigate long-term morbidity by facilitating early detection and timely intervention to reduce HF burden among cancer survivors.^10^

## Declarations

### Data Availability

No data were generated or analysed for this manuscript.
